# Rapid development of a mycotic aneurysm of the intracranial artery secondary to *Scedosporium apiospermum* sinusitis

**DOI:** 10.1016/j.mmcr.2016.11.001

**Published:** 2016-11-27

**Authors:** Yoshihiko Ogawa, Masatoshi Sato, Masato Tashiro, Masayuki Miyazaki, Kiyoshi Nagata, Nobuyuki Takahashi, Kei Kasahara, Koichi Izumikawa, Hisakazu Yano, Keiichi Mikasa

**Affiliations:** a Center for Infectious Diseases, Nara Medical University, Shijo 840, Kashihasa, Nara, Japan; b Department of Infectious Diseases, Nara City Hospital, Nara, Japan; c Department of Infectious Diseases, Nagasaki University Graduate School of Biomedical Sciences, Nagasaki, Japan; d Department of Neurology, National Center Hospital, National Center of Neurology and Psychiatry, Tokyo, Japan; e Department of Neurosurgery, Nara City Hospital, Nara, Japan; f Department of Neurology, Nara City Hospital, Nara, Japan; g Department of Microbiology and Infectious Diseases, Nara Medical University, Nara, Japan

**Keywords:** *Scedosporium apiospermum*, Mycotic aneurysm, Central nervous system infection

## Abstract

An 85-year-old man complained of a 2-month history of pain on the left side of his face. Brain computed tomography (CT) and magnetic resonance imaging/magnetic resonance angiography did not clearly show any intracranial abnormality and only showed fluid effusion in his left sphenoid sinus. Filamentous fungi were detected from the left sphenoid sinus specimen. The isolate was *Scedosporium apiospermum*. He was empirically treated with voriconazole, to which the isolate was susceptible. His consciousness decreased rapidly. Urgent 3D-CT angiography revealed an intracranial aneurysm near the left sphenoid sinus. Despite urgent coil embolization, the aneurysm ruptured, and he died.

## 1 Introduction

*Scedosporium apiospermum* is an ubiquitous filamentous fungus in the environment, found in the soil and polluted water. This organism can cause infections in not only immunocompromised hosts but also immunocompetent hosts. This organism has recently gained attention because of several reports about severe infections in tsunami survivors [1], [2], [3]. The identification of *S. apiospermum* is generally based on mycology culture, but colony formation usually takes time. Voriconazole is considered the first-choice drug against *S. apiospermum* infections [4]. Here, we describe a fatal case of central nervous system (CNS) infection with *S. apiospermum* in an immunocompetent patient empirically treated with voriconazole.

## 2 Case

An 85-year-old man complaining of a 2-month history of pain on the left side of his face was admitted to the department of neurosurgery (day 0). He was immunocompetent and was taking medication for hypertension. Non-steroidal anti-inflammatory drugs were prescribed at a local clinic 3 weeks before admission, but the pain did not improve. Five days before admission, blepharoptosis and diplopia occurred. On admission, his consciousness was clear. He was afebrile, and his blood pressure was high (166/76 mmHg) while medicated for his hypertension. Neurologic findings included pain in the V1 region of the left side of his face (but intact in V2/V3 regions) and left external ophthalmoplegia in all directions.

**Fig. 1:**
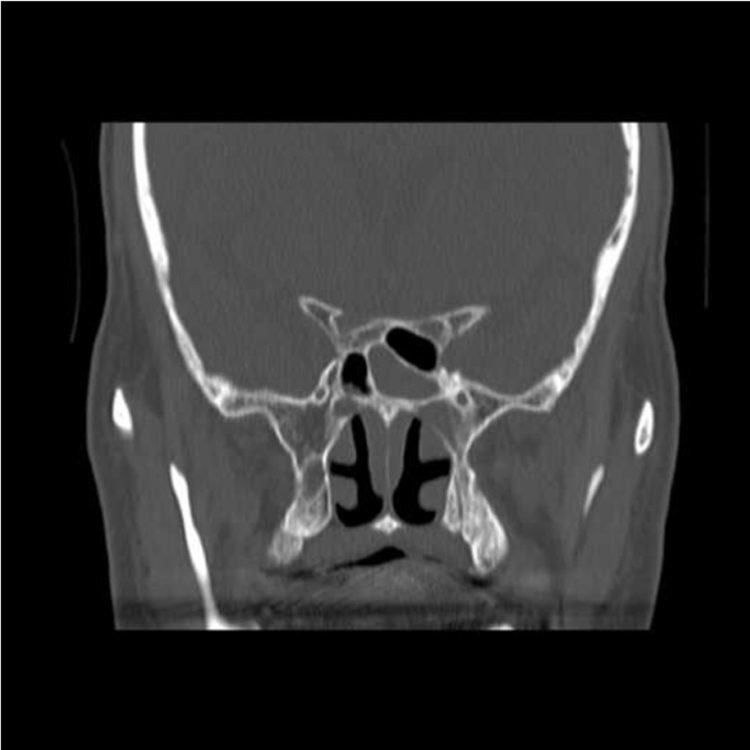
CT image showed fluid effusion in his left sphenoid sinus.

Brain computed tomography (CT) and magnetic resonance imaging (MRI)/magnetic resonance angiography (MRA) did not clearly show any intracranial abnormality and only showed fluid effusion in his left sphenoid sinus (Fig. 1). Therefore, he was transferred to the department of neurology. Peripheral blood tests showed elevated white blood cell count (16,860/µL) and C-reactive protein (6.38 mg/dL) and a mild increase in (1→3)beta-D-glucan (33.7 pg/mL). Lumbar puncture was performed, and increased first pressure (9 cmH2O), elevated protein (45 mg/dL), elevated cells (13/3 µL), and decreased sugar (70 mg/dL; sugar serum/cerebral spinal fluid (CSF) sugar, 0.79) were absent. The CSF culture was also negative. Therefore, we suspected orbital apex syndrome, and intravenous betamethasone therapy (12 mg/day) was started. His pain was relieved. However, upon tapering of the betamethasone dose, his pain returned. Brain MRI/MRA was performed, and the findings were similar to the previous results, with no intracranial abnormality. We also considered the pain from sinusitis. On admission day 8, absorption and biopsy examination of the nature osmium of the left sphenoid sinus was performed. Pus discharge was not discolored, and a small amount of serous discharge was obtained. At the same time, we resected part of the lumen mucosa for pathological examination. After the procedure, voriconazole (300 mg/12 h), ceftriaxone (2 g/day), and steroid pulse were administered intravenously. His pain improved, but remained.

**Fig. 2:**
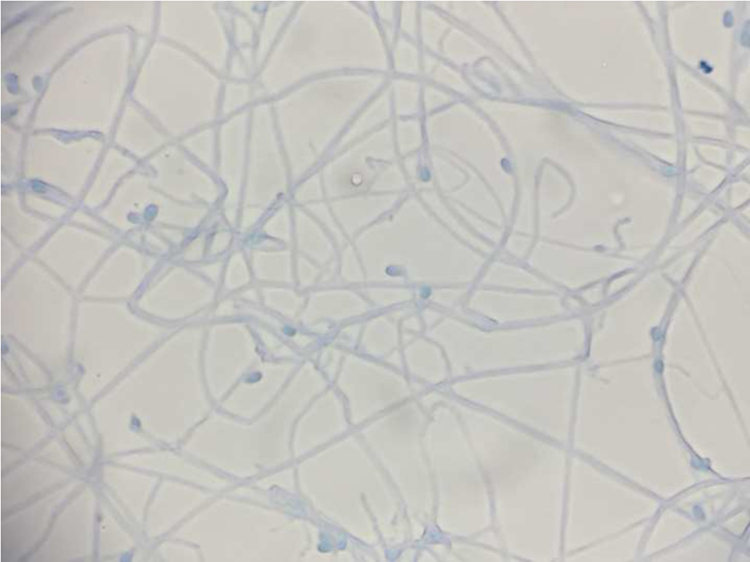
Lactophenol cotton blue stain, ×40.

A histopathologic examination revealed necrotic and granuloma tissue with invasion of lymphocytes and foamed cells, but the fungus was absent with Grocott and PAS staining; mycobacteria was also absent with Ziel-Nelsen staining. However, filamentous fungi were detected from the culture specimen, and the isolate was identified as *Scedosporium species* using macroscopic colony morphology and micromorphological characteristics (Fig. 2). We performed additional molecular analysis of the ribosomal internal transcribed spacer (ITS) and ribosomal large-subunit D1/D2 regions [5], [6] by amplifying sequences of 1078 bp and 584 bp, respectively. According to the BLAST database (http://blast.ddbj.nig.ac.jp/blastn?lang=ja), the ITS sequence of the isolate was 99% identical to *Scedosporium apiospermum* (accession number: KT323975), and the sequence of D1/D2 region was 99% identical to *S. apiospermum* (accession number: LT558764). Thus, we concluded that the isolate was *S. apiospermum.*

A drug susceptibility test was performed based on CLSI M38-A2 [7]. The minimum inhibitory concentrations (MIC) of amphotericin B, voriconazole, and itraconazole were 0.5 μg/mL, 0.5 μg/mL, and ≥8 μg/mL, respectively.

On admission day 19, he suddenly experienced a strong occipital headache. Thus, brain CT was performed, which showed a subarachnoid hemorrhage. At that time, his consciousness remained clear, and medical therapy for blood pressure control was primarily administered.

**Fig. 3:**
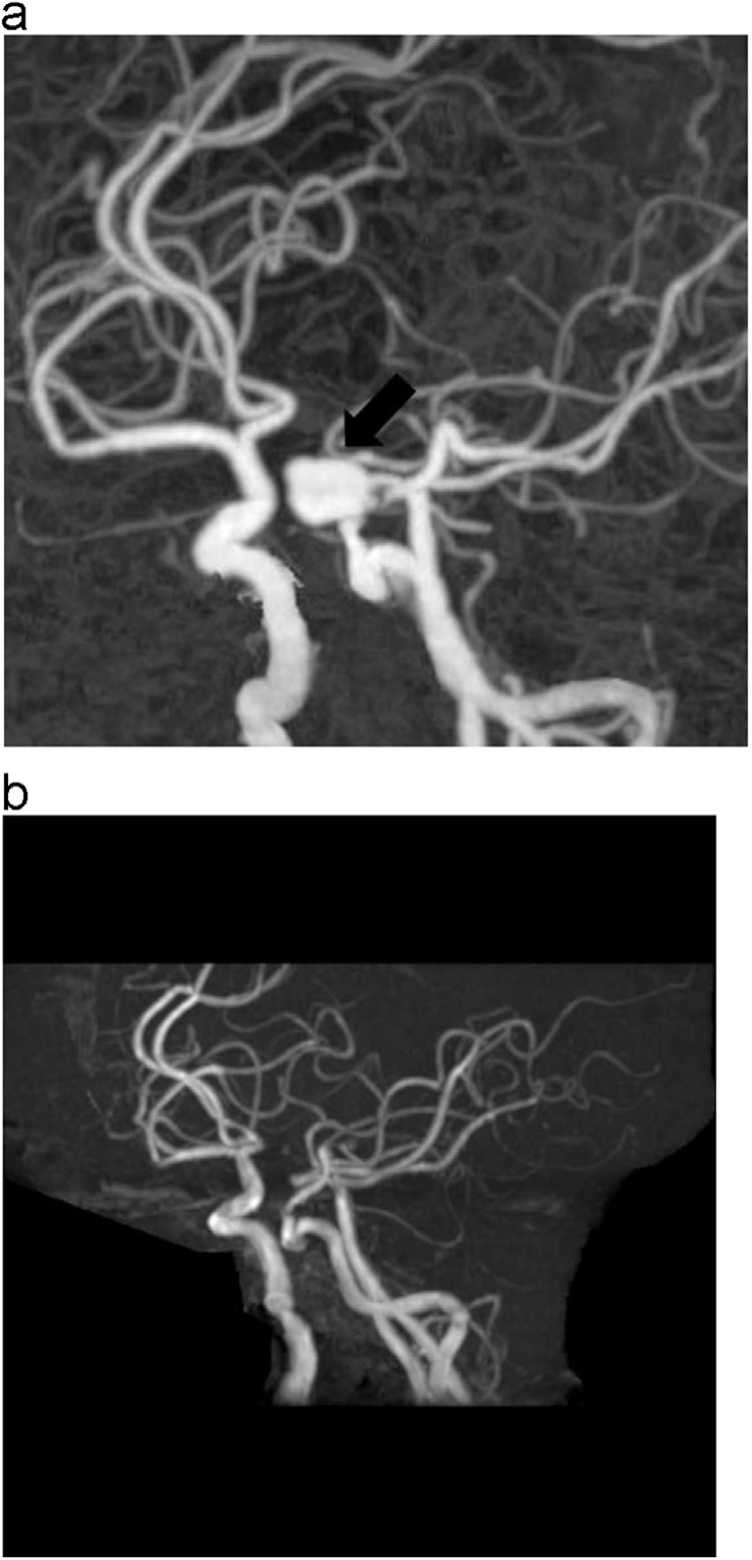
A. CTA revealed an intracranial aneurysm (14×14×15 mm) in proximity to the left sphenoid sinus (➡). B. The aneurysm was absent on the MRA two weeks previous.

On admission day 20, however, his consciousness decreased rapidly, and urgent 3D-CT angiography (CTA) revealed an intracranial aneurysm (14×14×15 mm) in proximity to the left sphenoid sinus, which was absent on the MRA two weeks previous (Fig. 3A and B). The osteoclastic image was not present. Therefore, on admission day 23, we performed coil embolization of the aneurysm. However, on the night of the same day, his blood pressure increased to 160 mmHg, and anisocoria developed. CT revealed rebleeding and brain swelling. Subsequently, his blood pressure declined suddenly, and cardiopulmonary arrest occurred. He was revived once with cardiopulmonary resuscitation, and he died on admission day 26.

## 3 Discussion

*S. apiospermum* is a filamentous fungus and was first described as a cause of mycetoma over 100 years ago. High levels of genetic variation within this species were recently demonstrated [8]. Human infection often results from inhalation of spores from the environment into the lungs or paranasal sinuses or through direct inoculation [9].

Although the identification of *S. apiospermum* is generally based on mycology culture, the usefulness of several gene assays has been reported [10], owing to the delay in colony formation. In the present case, we demonstrated the usefulness of the molecular identification. The usefulness of Matrix Assisted Laser Desorption Ionization-Time of Flight (MALDI-TOF) for the identification of this organism was also recently reported and could represent the next-generation identification method [11].

High mortality rates with CNS infection was recently reported based on the review of about 99 case series of CNS infection with *S. apiospermum* [12]. Eleven of 12 cases of mycotic aneurysm described in the literature died [13]. Similar to the present case, Watson et al. described an aneurysm of the basilar artery from sinusitis [14]; the patient died despite surgical treatment and the administration of several antifungal agents (fluconazole, miconazole, and amphotericin B). We empirically used voriconazole, which is considered first-line treatment against *S. apiospermum* infections [4], at an early stage, and the strain tested susceptible to voriconazole; however, the infection was not controlled. The steroid use for his various CNS symptoms or failure of the voriconazole concentration within the therapeutic range owing to the interactions with the other drugs might explain the inability to control the infection; we did not perform therapeutic drug monitoring for voriconazole. However, CNS infections with this organism are reportedly fatal, as discussed earlier in this paragraph.

Regarding the drug susceptibility test results for the isolate, the MICs of itraconazole and voriconazole were typical and that of amphotericin B was not. Lackner et al. described the MIC distribution of 154 strains of *S. apiospermum* [15]; for the major population, the MICs of itraconazole, voriconazole, and amphotericin-B were>16 μg/mL, 0.5–2.0 μg/mL, and 8.0 to >16 μg/mL, respectively. Thus, we repeatedly performed drug susceptibility tests, and reproducibility was obtained.

In summary, CNS infection and aneurysm due to *S. apiospermum* are rare, but might be increasing; the disease is extremely fatal. Optimal treatment remains unknown; therefore, this disease should be diagnosed and treated as early as possible, utilizing techniques such as the ITS gene assay.

## Conflict of interest

There are none.
